# An evaluation of marine regions relevant for ocean color system vicarious calibration

**DOI:** 10.1016/j.rse.2016.11.020

**Published:** 2017-03-01

**Authors:** Giuseppe Zibordi, Frédéric Mélin

**Affiliations:** European Commission, Joint Research Centre, Ispra, Italy

**Keywords:** Ocean color, System vicarious calibration

## Abstract

System Vicarious Calibration (SVC) is the fundamental process commonly implemented to meet uncertainty requirements in satellite ocean color data. It is performed by applying gain factors, *g*-factors, to the pre-launch calibration coefficients of the space sensor already corrected for sensitivity decay with time. Mission specific *g*-factors are determined from top-of-the-atmosphere data computed by propagating highly accurate in situ values of the water-leaving radiance, *L*_w_, to the satellite sensor. Values of *L*_w_ from marine regions characterized by oligotrophic/mesotrophic waters and maritime aerosols, high environmental stability and spatial homogeneity, low cloudiness and absence of any source of land contamination, are essential to determine *g*-factors applicable to the creation of Climate Data Records (CDRs) from multiple ocean color missions. Accounting for the location of existing and potential new SVC fixed sites, marine regions satisfying SVC requirements for the generation of CDRs have been identified through the analysis of satellite data from recent ocean color missions.

## Highlights

• System Vicarious Calibration (SVC) for ocean color missions
• Climatology of marine and atmospheric optical properties of regions relevant for SVC
• Recommendations for new SVC regions

## 1 Introduction

System Vicarious Calibration (SVC) is the indirect calibration of satellite ocean color sensors that minimizes the combined effects of atmospheric correction and sensor calibration uncertainties on derived radiometric data. SVC is performed to meet uncertainty requirements in data products such as the spectral water leaving radiance *L*_w_ determined from the top-of-atmosphere radiance *L*_T_ (Gordon, 1987, Gordon, 1998): it is accomplished by applying gain factors, *g*-factors, to pre-launch spectral calibration coefficients already corrected for sensitivity change with time (e.g., Eplee et al., 2001, Franz et al., 2007, Werdell et al., 2007, Bailey et al., 2008, Mélin and Zibordi, 2010).

Values of *g*-factors are determined by the ratio of simulated to measured top-of-the-atmosphere spectral *L*_T_ values, where the simulated ones are derived by propagating accurate in situ *L*_w_ to the satellite level. Unique to SVC is the use of the same models and algorithms embedded in the atmospheric correction process for the determination of satellite-derived radiometric data. Thus SVC is a relative radiometric calibration specific for each mission, i.e., for each ocean color sensor and atmospheric correction framework.

It is emphasized that SVC implies availability of highly accurate in situ *L*_w_ data in the visible spectral region. This is as opposed to the near-infrared bands where modeled *L*_T_ values with uncertainties up to a few percent (which may imply extremely high relative uncertainties in the corresponding *L*_w_) do not significantly affect the SVC process (Wang and Gordon, 2002).

In addition to the accuracy of in situ *L*_w_ data, a number of features specific to the measurement site such as small environmental variability (i.e., a high intra-annual stability), high spatial homogeneity, mesotrophic/oligotrophic waters, maritime aerosols and lack of any land perturbation (Zibordi et al., 2015), are also fundamental requirements for ocean color SVC supporting climate change applications. This implies that not all individual in situ measurements or series of measurements, regardless of their level of accuracy, meet SVC needs for the construction of Climate Data Records (CDRs) from multiple ocean color missions.

The objective of this study is to identify marine regions satisfying SVC requirements for the construction of CDRs. By using time-series of satellite ocean color global data products, the study investigates the fulfilment of the requirements mentioned above for a number of regions already hosting SVC fixed sites or for which new sites are under consideration.

This work adds to ongoing investigations like those on data merging (e.g., Maritorena et al., 2010) or on the effects of biases affecting independent missions (e.g., Mélin, 2016), all contributing to the international effort to create ocean color CDRs by benefitting from global long-term missions such as the Joint Polar Satellite System (JPSS) from the National Oceanic and Atmospheric Administration (NOAA) started in 2011, Sentinel-3 from the European Space Agency (ESA) started in 2016, the Global Change Observation Mission-Climate (GCOM-C) from the Japan Aerospace Exploration Agency (JAXA) scheduled from 2017, and the Plankton Aerosols Clouds and ocean Ecosystems (PACE) from the National Aeronautics and Space Administration (NASA) scheduled from 2022.

## 2 Background

The water-leaving radiance *L*_w_ is the primary satellite-derived radiometric quantity from which high-level data products such as the remote sensing reflectance *R*_rs_ or chlorophyll-a concentration *Chla* are determined. This has led to the inclusion of *L*_w_ among the oceanic Essential Climate Variables (ECV) in association with uncertainty requirements of 5% in the blue-green spectral regions and radiometric stability better than 0.5% per decade (WMO, 2011). SVC is the technique commonly used to address such requirements. However, while the 5% uncertainty can be met with moderate efforts using alternative sources of in situ data, the 0.5% stability requirement is only achievable at the expense of extraordinary efforts through the application of state of the art radiometry and at sites exhibiting high intra-annual stability and spatial homogeneity of marine and atmospheric optical properties (Zibordi et al., 2015). This comprehensive framework is required by the need to ensure the same high precision to *g*-factors determined for successive missions. In fact, changes with time of uncertainties characterizing in situ measurements or observation conditions, may affect the precision of *g*-factors determined during the different time intervals of independent missions. This need for high precision ultimately favors SVC sites exhibiting: *i*. a high spatial homogeneity that minimizes the impact of the different geometric resolutions characterizing in situ and satellite observations; and *ii*. a high intra-annual stability of the marine and atmospheric optical properties that minimizes uncertainties due to the varying performance of the atmospheric correction process across different observation conditions. It must be additionally noted that a high intra-annual stability is commonly associated with low concentrations of seawater optically significant constituents typical of oligotrophic waters (Iverson et al., 2000). This implies a low bio-optical complexity that improves modeling accuracy (e.g., while removing the effects of the non-isotropic distribution of the in-water light field in satellite data to match satellite and in situ viewing geometries) and that consequently increases the precision of *g*-factors.

Overall, general requirements for in situ data supporting SVC for ocean color climate applications (see Zibordi et al., 2015) are summarized by the need for long-term, hyperspectral, traceable and highly accurate measurements performed at sites:

1. Located in a region chosen to maximize the number of high-quality matchups by trading off factors such as best viewing geometry, sun-glint avoidance, low cloudiness, and additionally set away from any continental contamination and at a distance from the mainland to safely exclude adjacency effects in satellite data;
2. Exhibiting known or accurately modelled optical properties coinciding with maritime atmosphere and oligotrophic/mesotrophic waters, to represent the majority of world oceans and minimize relative uncertainties in computed *g*-factors;
3. Characterized by high spatial homogeneity and small environmental variability, of both atmosphere and ocean, to increase precision of computed *g*-factors.

It is mentioned that the work by Zibordi et al. (2015) indicates that the creation of CDRs from independent ocean color missions should ideally rely on the application of the same atmospheric correction process and on time-series of in situ *L*_w_ data from a single reference SVC site. However, the work also recognizes that strategies to support long-term climate investigations recommend redundancy of in situ SVC measurement sites (IOCCG, 2012). This implies establishing multiple SVC sites: i. relying on in situ radiometry systems equivalent in terms of data accuracy and long-term performance; ii. and located in regions also exhibiting ideal and likely similar measurement conditions.

The high cost of establishing and maintaining over decades SVC sites meeting the requirements for the creation of CDRs from multiple ocean color missions, nevertheless, suggests a careful evaluation of suitable marine regions without neglecting the fundamental necessity to benefit from logistical support from infrastructures located at nearby islands or coastal locations.

## 3 Regions, data and methods

### 3.1 Marine regions

As already anticipated, the regions considered in this analysis (see Table 1), are those related to fixed sites already in use for ocean color SVC or alternatively potential SVC sites under consideration because of their atmospheric and marine optical properties expected to be representative of the world oceans.

The regions hosting established SVC sites include: the North Pacific Ocean (NPO) with the Marine Optical Buoy (MOBY) site managed by the US National Oceanic and Atmospheric Administration (NOAA; Clark et al., 1997, Clark et al., 2002, Clark et al., 2003); the Arabian Sea (ASea) with the Kavaratti Site managed by the Indian Space Research Organization (ISRO; Shukla et al., 2013); the Ligurian Sea (LSea) with the BOUée pour l'acquiSition d'une Série Optique à Long termE (BOUSSOLE) site managed by the French Laboratoire d'Océanographie de Villefranche (LOV; Antoine et al., 2008b). The regions for which the setting up of new SVC sites has been a matter of discussion within the scientific community comprise: the Mediterranean Sea (MSea) near the Island of Crete; the Caribbean Sea (CSea) near Puerto Rico Islands; the North Atlantic Ocean (NAO) near Azores Islands; and the Eastern Indian Ocean (EIO) near Rottnest Island off Perth. In addition to the previous regions, the South Pacific Gyre (SPG) is also included as a virtual reference region due to its highly oligotrophic waters and its expected high temporal stability (Twardowski et al., 2007).

It is noted that the considered regions are characterized by Case-1 waters (i.e., exhibit optical properties that can be described as a function of *Chla*, only), which are representative of the most common oceanic waters. It is also pointed out that all regions, with the exception of the virtual SPG one, are located nearby islands or coastal locations favouring maintenance services of the offshore SVC measurement infrastructure, but also at distances from the coast minimizing land contamination such as adjacency effects in satellite data (Bulgarelli et al., 2014).

It is finally recognized that the regions included in this study are not likely to reflect all those potentially suitable for ocean color SVC. Still, not excluding alternatives, the regions considered provide an overview of the marine/atmospheric optical properties of those potential SVC sites currently considered of major relevance to support the creation of ocean color CDRs.

### 3.2 Remote sensing data and methods

The accuracy of ocean color data products is related to a number of factors encompassing the overall calibration of the space sensor and atmospheric correction scheme applied in conjunction with the embedded marine/atmospheric models and algorithms. These factors may certainly lead to the generation of data products with uncertainties varying from region to region as a function of different marine/atmospheric optical properties or observation/illumination geometries (Mélin et al., 2016).

The Sea-Viewing Wide Field-of-View Sensor (SeaWiFS, Hooker et al., 1992) ocean color data products, besides constituting one of the longest time-series from a single mission, are among those most investigated and exploited. In particular they benefitted from a number of incremental improvements in data processing and related models/algorithms (e.g., Gordon and Wang, 1994, Wang et al., 2005, Franz et al., 2007, Ahmad et al., 2010, Hu et al., 2012b), and additionally were the foundation of extensive and geographically distributed validation exercises for radiometric and derived marine products (e.g., Gregg and Casey, 2004, Mélin et al., 2005, Bailey and Werdell, 2006, Zibordi et al., 2006, Antoine et al., 2008a, Hu et al., 2013) as well as for the aerosols (e.g., Wang et al., 2005, Mélin et al., 2013a, Mélin et al., 2013b). Specifically, Hu et al. (2013) and Mélin et al. (2016) confirmed the capability of SeaWiFS to meet the 5% uncertainty requirement in the blue bands in oligotrophic waters, even though often reaching values of 10–15% in the green bands.

The previous elements indicate confident applicability of SeaWiFS marine/atmospheric data products to open sea investigations (e.g., Gregg et al., 2003, McClain et al., 2004, Gregg, 2008, Vantrepotte and Mélin, 2011). Thus, by relying on this evidence, the analysis on the atmospheric and marine bio-optical properties of the regions included in this study is carried out with data from the SeaWiFS mission (1997–2010) with the assumption that any geographically dependent uncertainty does not affect basic findings.

The following analysis is performed using SeaWiFS Level-2 daily 1-km spatial resolution and Level-3 monthly averages 24th-degree spatial resolution products, both from the R2014.0 reprocessed data distributed by the US National Aeronautics and Space Administration (NASA).

Time-series of monthly averages of atmospheric and marine data products are applied to investigate the climatology of atmospheric/marine bio-optical properties. The list of these quantities is presented in Table 2: i. *R*_rs_ relevant to characterize the water type associated with each region and to address the impact of the in situ radiometric signal in the uncertainty of *g*-factors; ii. diffuse attenuation coefficient at 490 nm, *K*_d_(490), and concentration of chlorophyll-*a*, *Chla*, relevant to discuss the climatology of marine bio-optical properties; iii. aerosol optical thickness at 865 nm, τ_a_(865), and the Ångström exponent, α, relevant to discuss the climatology of atmospheric optical properties.

Instead of monthly averages, time series of daily full resolution *R*_rs_ and derived data products are used to evaluate the potential of each region to contribute to the construction of in situ and satellite matchups for ideal observation conditions (e.g., when exhibiting high spatial homogeneity and not affected by clouds, high glint, high viewing angle). In view of discussing the effects of different viewing geometries, analyses are also extended to data from a number of ocean color sensors, all processed with the same system (i.e., the SeaWiFS Data Analysis System (SeaDAS) version 7.2 or above).

## 4 Analysis of monthly averaged data

This section aims at providing a comprehensive overview of the marine and atmospheric optical properties for the various regions included in the study. The climatology of relevant optical properties has been determined using mean values from monthly averages of the 5 × 5 data elements centered at each region included in the analysis (the use of mean instead of the alternative median, ensures consistency with the input Level-3 monthly averages). Data have been retained when at least one of the data elements exhibits a valid value.

### 4.1 Climatology of marine bio-optical properties: *R*_rs_ spectra and time-series of *R*_rs_(555), *K*_d_(490) and *Chla*

**Fig. 1:**
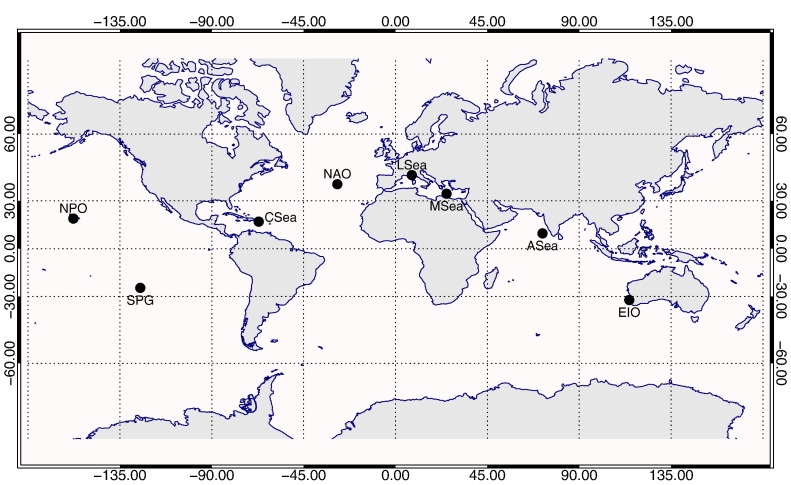
Map of the marine regions of interest (see Table 1 for details).

**Fig. 2:**
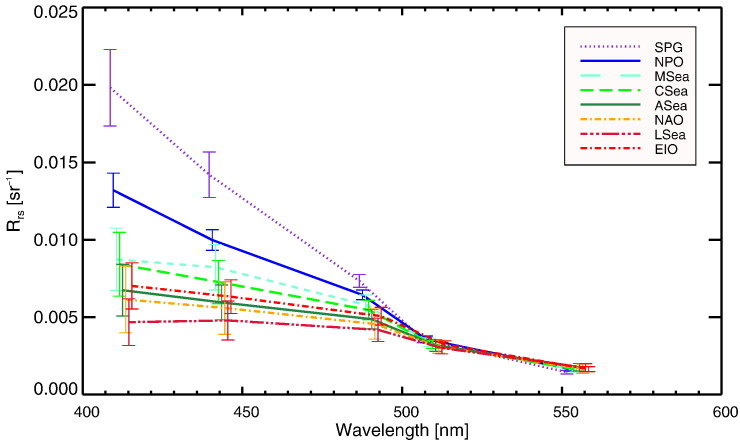
Mean values of *R*_rs_ determined from the entire SeaWiFS mission at the 412–555 nm bands for the considered marine regions. Error bars indicate ± 1σ. Spectra are incrementally shifted by 2 nm to increase readability of the figure while values at the 670 nm band, which are almost nil for all the regions, are not plotted.

Mean *R*_rs_ spectra and standard deviations determined over the entire SeaWiFS mission are presented for the different marine regions identified in Fig. 1. Spectra show a range of cases varying from those representative of oligotrophic waters to those typical of mesotrophic waters (see Fig. 2). The highest values are found in the oligotrophic waters of the South Pacific Gyre (SPG) and of the North Pacific Ocean (NPO). On average the lowest *R*_rs_ spectra are found in the Ligurian Sea (LSea) and North Atlantic Ocean (NAO), while slightly higher *R*_rs_ are observed for the Arabian Sea (ASea) and Eastern Indian Ocean (EIO) waters. At 412 nm, the *R*_rs_ values of the Mediterranean Sea (MSea) and Caribbean Sea (Csea) are approximately twice that of the LSea mesotrophic region.

Standard deviations σ of *R*_rs_ in Fig. 2 largely vary from region to region and are likely explained by seasonal cycles. Despite the bluest waters, SPG exhibits values of σ much higher than those determined for NPO.

Notable is also the difference in the slope of *R*_rs_ in the blue spectral interval at MSea with respect to the other oligotrophic regions. This feature that also characterizes the LSea mesotrophic waters, is confirmed by field measurements (Zibordi et al., 2011). Explanation is likely given by the presence of an excessive amount of yellow substance in the Mediterranean Sea waters with respect to comparable oceanic areas (Morel and Gentili, 2009). An alternative hypothesis is the presence of submicron Saharan dust that increases absorption in the blue and backscattering in the green parts of the spectrum (Claustre et al., 2002).

It is noted that radiometric data from mesotrophic rather than oligotrophic waters, minimize uncertainties in *g*-factors when using an equivalent number of in situ data and assuming comparable uncertainties (see discussion in Zibordi et al., 2015). This may suggest preference for mesotrophic rather than oligotrophic regions. However, the more advantageous *L*_w_ spectral values obtainable in mesotrophic waters may be outclassed by the higher intra-annual stability and spatial homogeneity typical of oligotrophic waters, both creating observation conditions favoring precision of *g*-factors.

**Fig. 3:**
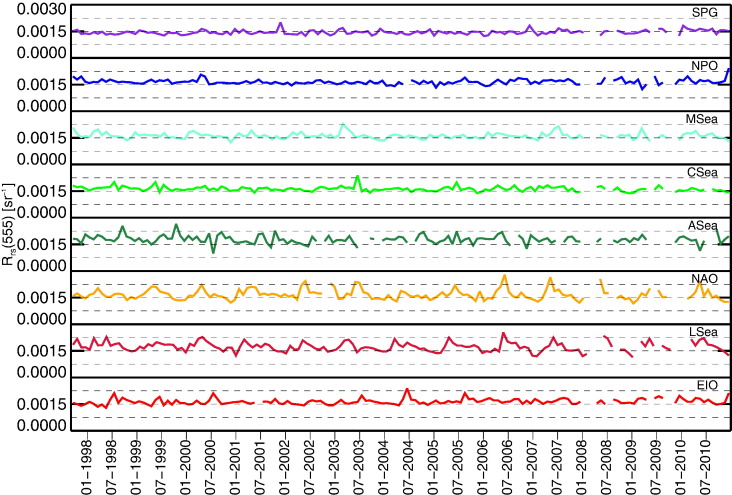
Time-series of monthly values of *R*_rs_(555) for the various regions of interest.

**Fig. 4:**
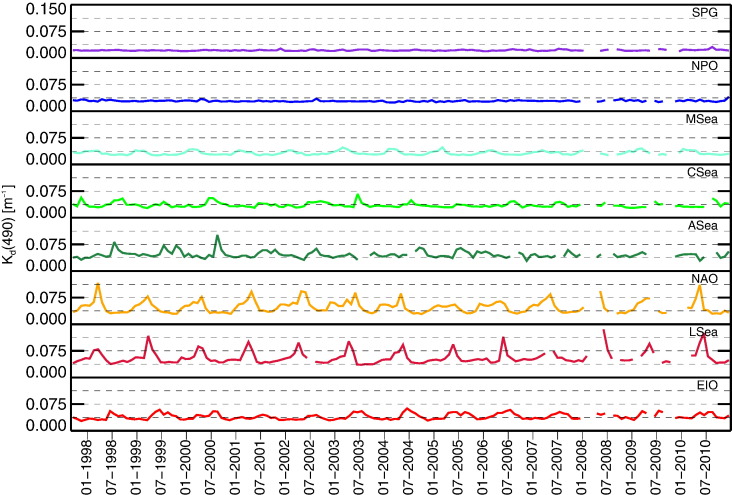
Time-series of monthly values of *K*_d_(490) for the various regions of interest. Increase of *K*_d_(490) in Case-1 waters indicates increase in *Chla*.

**Fig. 5:**
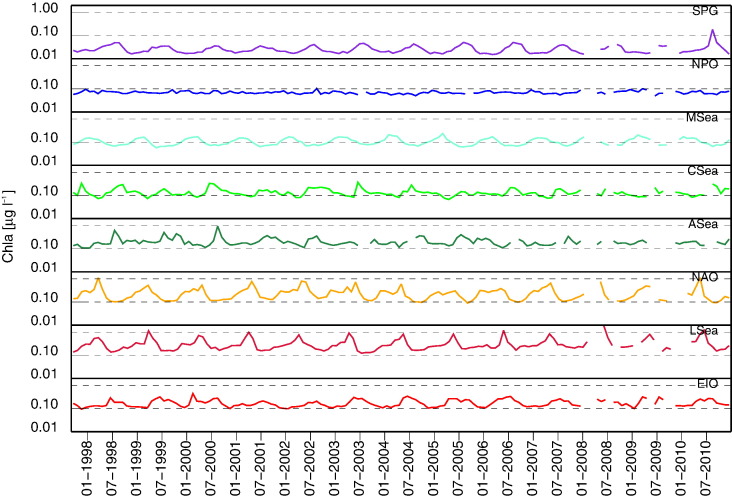
Time-series of monthly values of *Chla* (in logarithmic scale) for the various regions of interest.

Fig. 3, Fig. 4, Fig. 5 show the time-series of *R*_rs_(555), *K*_d_(490) and *Chla* for the regions considered. While the quantities *K*_d_(490) and *Chla* are bio-optical indicators suitable to evaluate the intra-annual variability of the region, *R*_rs_ at the 555 nm band is included to investigate the existence of potential uncorrelated changes in the concentration of optically significant constituents. This specific capability is offered by the small dependence of *R*_rs_(555) on *Chla* in Case-1 waters (see the slight differences among *R*_rs_(555) displayed in Fig. 2 for the various marine regions representing different bio-optical regimes).

The time-series of *R*_rs_(555) show the highest intra-annual stability for SPG, NPO and CSea regions with standard deviations σ of 0.12–0.14 × 10^− 3^ sr^− 1^. Larger changes are observed for ASea, LSea and NAO with values of σ in the range of 0.24–0.30 × 10^− 3^ sr^− 1^. Intermediate values with σ = 0.16 × 10^− 3^ sr^− 1^ are shown by MSea and EIO. However, it could be that part of the observed variability is produced by seasonal changes in illumination conditions (i.e., changes in θ_0_) not fully removed by the atmospheric correction process.

The time-series of *K*_d_(490) also show the lowest and most stable values for the SPG and NPO regions. Specifically, SPG exhibits mean value of 0.022 m^− 1^ and NPO of 0.029 m^− 1^, both with σ = 0.002 m^− 1^. Close values are shown by MSea with a mean of 0.032 m^− 1^ and σ = 0.005 m^− 1^, and also by CSea with a mean of 0.036 m^− 1^ and σ = 0.007 m^− 1^. Higher values characterize EIO, ASea and NAO with a mean in the range of 0.040–0.048 m^− 1^ and σ in the range of 0.008–0.017 m^− 1^. LSea exhibits the largest values with a mean of 0.056 m^− 1^ and σ = 0.018 m^− 1^.

Consistent with *K*_d_(490), *Chla* time-series exhibit the lowest and most stable values for SPG and NPO with means of 0.03 and 0.07 μg l^− 1^, respectively, and σ of 0.01–0.02 μg l^− 1^. In agreement with the oligotrophic nature of the Eastern Mediterranean basin (e.g., Bosc et al., 2004), MSea also exhibits low and relatively stable values with mean of 0.11 μg l^− 1^ and σ = 0.04 μg l^− 1^. Larger values characterize the CSea and EIO regions with means of 0.14 and 0.18 μg l^− 1^, respectively, both with σ = 0.06 μg l^− 1^. The largest values are then observed for ASea, NAO and LSea with means in the range of 0.21–0.33 μg l^− 1^ and σ of 0.10–0.25 μg l^− 1^.

**Fig. 6:**
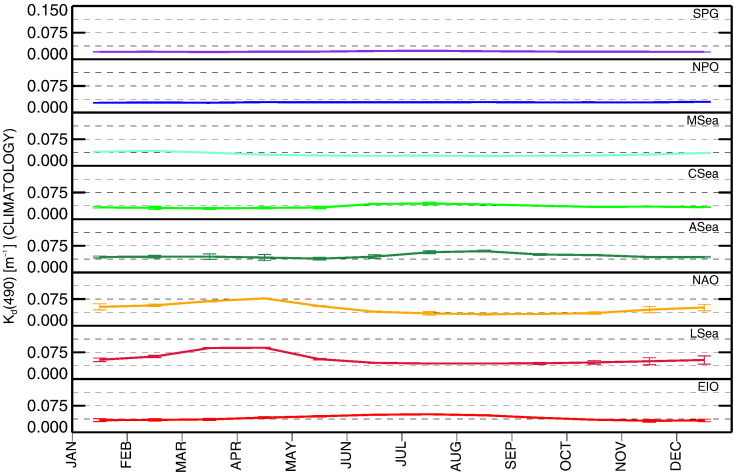
Monthly climatology of *K*_d_(490) for the various regions of interest.

Fig. 6 displays the annual climatology of *K*_d_(490). LSea and NAO exhibit the highest seasonal variability with σ of 0.016 and 0.015 m^− 1^, respectively. SPG and NPO show the lowest, with σ of approximately 0.001 m^− 1^. All the other regions exhibit values of σ in the range of 0.005–0.007 m^− 1^.

In summary, excluding SPG, unequivocally NPO exhibits the least pronounced annual cycles and the clearest waters. Additional regions that also exhibit a low intra-annual variability of bio-optical properties are MSea and CSea. Among these, MSea is characterized by the lowest mean values and seasonal variability for both *K*_d_(490) and *Chla*. The largest mean values and variabilities for both *K*_d_(490) and *Chla* are observed for LSea.

### 4.2 Climatology of atmospheric optical properties: time-series of τ_a_(865) and α

**Fig. 7:**
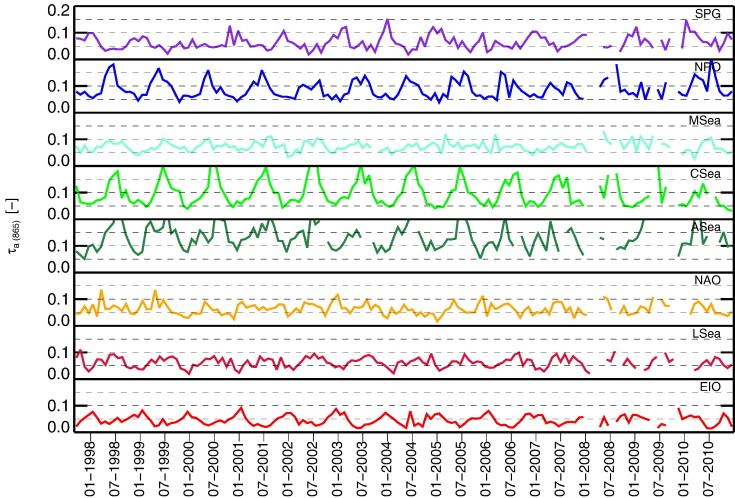
Time-series of monthly values of τ_a_(865) for the various regions of interest.

**Fig. 8:**
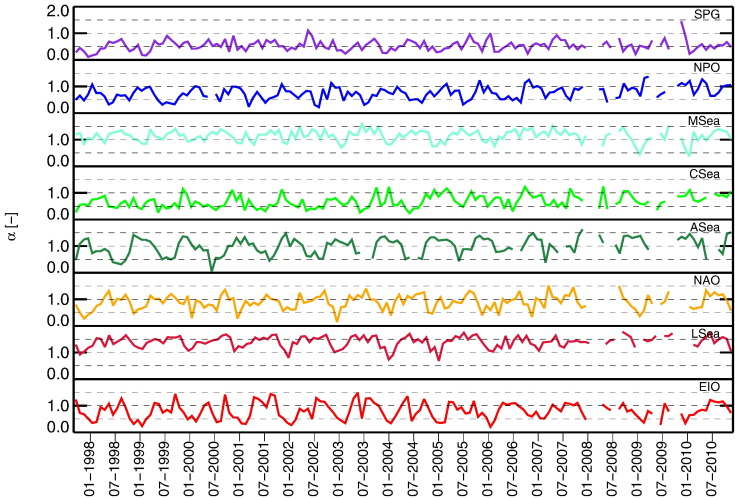
Time-series of monthly values of α determined from near-infrared spectral bands for the various regions of interest.

The atmospheric optical properties are illustrated in Fig. 7, Fig. 8 through τ_a_(865) that provides information on the aerosol load, and through α chosen to describe the aerosol type. The lowest mean values and seasonal variabilities of τ_a_(865) are those determined at EIO, LSea, NAO, SPG and MSea with means in the range of 0.05–0.07 and σ of 0.02–0.03 (admittedly, the relative values of τ_a_ may change between sites at shorter wavelengths as a function of α). Slightly higher temporal variabilities are noted for NPO with mean value of 0.09, and σ = 0.03. The highest values are observed for CSea and ASea with means of 0.10 and 0.13, respectively, both with σ of 0.05. The higher summer values at CSea are consistent with an influx of dust aerosols crossing the Atlantic Ocean towards the Caribbean Sea with possible contributions from biomass burning in South America (e.g., Colarco et al., 2003, Prospero et al., 2014, Yu et al., 2015).

Looking at α, the SPG, CSea and NPO regions exhibit mean values in the range of 0.5–0.8, with σ of 0.20–0.24, coherent with dominant marine aerosols (Smirnov et al., 2002, Smirnov et al., 2003, Smirnov et al., 2009). Slightly higher values are observed for EIO, NAO, and ASea with means in the range of 0.80–0.99 and σ of 0.28–0.32. Over a background of marine aerosols, the region of the Azores (i.e., NAO) can be subject to episodic influx of African desert dust (Chazette et al., 2001), while the annual cycle observed at ASea is coherent with the oscillations affecting the Arabian Sea (see Holben et al., 2001 for the Maldives) with summer monsoon events leading to low α due to aerosols dominated by sea-salt and dust (Vinoj and Satheesh, 2003), and winter monsoon events leading to high α determined by aerosols from the Indian sub-continent (Ramanathan et al., 2001). The highest values but also the lowest seasonal variabilities are observed at MSea and LSea with means of 1.14 and 1.40, respectively, both with σ = 0.22. These values indicate aerosol significantly affected by continental origin at LSea and to a lesser extent at MSea. Actually, all the Mediterranean Sea is under the influence of aerosols from diverse sources, including marine, continental of various types, desert dust and biomass burning (Lelieveld et al., 2002, Pace et al., 2006, Sciare et al., 2008). It is mentioned that the values of α for MSea are coherent with field measurements performed at the Island of Crete (Bryant et al., 2006, Kalivitis et al., 2007). However, annual means of field observations (Smirnov et al., 2009) as well as validation statistics (Mélin et al., 2013b) suggest that α determined from SeaWiFS data might be somewhat overestimated around Rottnest Island (EIO).

**Fig. 9:**
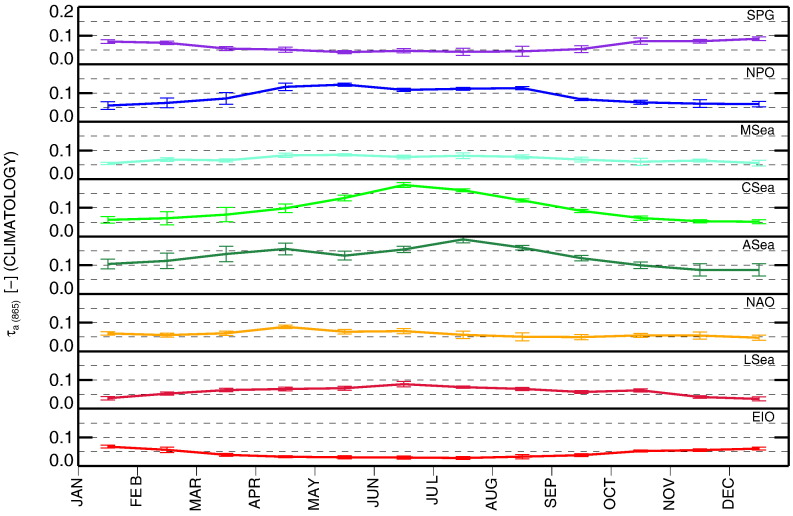
Monthly climatology of τ_a_(865) for the various regions of interest.

**Fig. 10:**
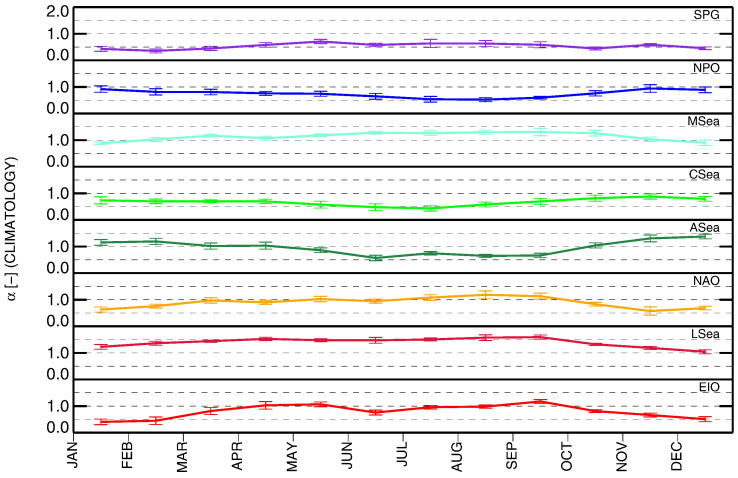
Monthly climatology of α for the various regions of interest.

The annual climatology of τ_a_(865) is illustrated in Fig. 9. It indicates the highest intra-annual variability for CSea with σ = 0.045 and the lowest for NAO with σ = 0.011. When looking at the annual climatology for α displayed in Fig. 10, LSea, CSea, NPO and MSea show the lowest variability with σ in the range of 0.11–0.15. Differently, ASea and EIO exhibit the highest values with σ of 0.25 and 0.27, respectively.

Overall, excluding SPG, the lowest and most stable values of τ_a_(865) are observed at NAO, EIO, MSea and LSea. In contrast, again excluding SPG, the regions showing dominance of maritime aerosol characterized by low α and intra-annual variability are CSea (with exceptions in summer) and NPO.

### 4.3 Illumination: θ_0_

**Fig. 11:**
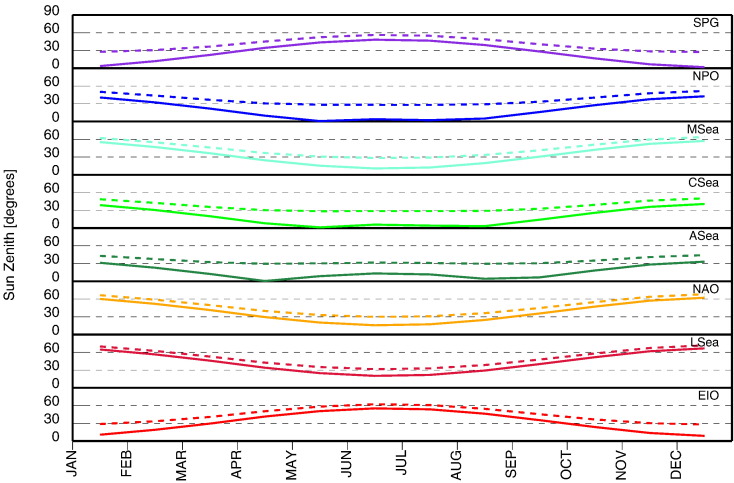
Intra-annual variations of θ_0_ at the regions of interest. The continuous lines indicate the values of θ_0_ at local noon, while the dashed lines indicate the values of θ_0_ at approximately ± 2 h from local noon.

Seasonal changes in the illumination conditions are illustrated in Fig. 11 through values of the sun zenith angle θ_0_. The continuous lines indicate θ_0_ at local noon, while the dashed lines indicate values at approximately ± 2 h from local noon chosen to cover a realistic interval of satellite overpass times. As expected, in agreement with latitude values, the lowest annual changes in θ_0_ at local noon are observed for the ASea, CSea and NPO regions with values within 2–43°. Values of θ_0_ in the range of 9–57° characterize EIO and MSea, while LSea and NAO exhibit values in the range of 16–67°.

These data indicate that the geometrical component of illumination (i.e., θ_0_) is an additional source of optical stability for sites like MOBY or Kavaratti. Large changes in θ_0_ may certainly decrease precision of *g*-factors and become the source of differences among SVC sites. However, it must be also recognized that large variations in θ_0_ may offer the capability to comprehensively and systematically investigate the effects of illumination conditions and consequently of bi-directional effects, as needed to improve data processing by minimizing related sources of uncertainty.

## 5 Analysis of daily full-resolution data products

In order to investigate the characteristics of the study regions in more detail and particularly to address their potential for match-up collection, the analysis is extended to SeaWiFS Level-2 full resolution daily data (so-called Local Area Coverage data). Additionally, to discuss matchup rates as a function of mission-specific features (orbit, overpass time, width of the viewing swath, …), statistical analyses are also performed with Level-2 data from the Moderate Resolution Imaging Spectroradiometer (MODIS) onboard the Aqua platform, MEdium Resolution Imaging Spectrometer (MERIS) onboard the Envisat platform, and Visible Infrared Imaging Radiometer Suite (VIIRS) onboard the Suomi National Polar-Orbiting Partnership (Suomi NPP) spacecraft. The period of analysis is 5-year (limited to 4 for VIIRS) typical of mission lifetimes, specifically 1999–2003 for SeaWiFS, 2003–2007 for MODIS and MERIS, and 2012–2015 for VIIRS.

The SPG site is excluded from this analysis due to the relatively low number of full resolution data available for SeaWiFS and additionally by the difficulty to establish an SVC site in the region.

### 5.1 Cloud, high glint and high viewing angle flags

Effects of a number of exclusion flags determined by SeaDAS are investigated to discuss the probability of gathering high quality matchups of satellite and in situ data at the marine regions considered. The analysis indicates that flags responsible for at least 90% of the exclusions incurred by the application of all SeaDAS Level-2 default flags (see http://oceancolor.gsfc.nasa.gov/cms/atbd/ocl2flags), are those for screening cloud/ice contamination, high glint perturbations and high viewing angles effects. In the following analysis the limit for the satellite zenith angle is set to 60° (equal to the current SeaDAS default value). It is however mentioned that early SVC exercises were performed with an angle limit of 56° (Franz et al., 2007) and that a recent assessment of satellite derived *R*_*rs*_ indicated zenith angle dependences above 40° (Barnes and Hu, 2016).

Note that the three main flags applied in the following analysis are largely independent of the algorithms associated with the atmospheric correction process: they specifically depend on geometry (viewing angle), geometry and wind (glint) and on conservative tests applied to top-of-atmosphere Rayleigh-corrected radiance for cloud screening (which may be additionally triggered by bright waters or thick aerosol plumes (Banks and Mélin, 2015)). These elements attribute relevance to the resulting statistics beyond the application of any specific atmospheric correction scheme.

It is noted that high viewing angles and, to a lesser extent glint perturbation, are related to the instrument design and not solely to regional geophysical features. Still, the analysis of high glint and high viewing angle flagged data is considered relevant to illustrate typical cases directly applicable to future missions. In fact, it is evident that for matchup collection, MERIS is hindered by the smaller viewing swath (i.e., 1150 km) and the lack of tilt capability when compared to SeaWiFS having a larger swath (i.e., 2800 km) and a tilted view to reduce the probability of glint conditions (Gregg and Patt, 1994).

Considering that satellite observations are rejected if at least one of the 5 × 5 Level-2 elements centered at the considered region is affected by the specific exclusion flag(s), results from the analyses of SeaWiFS, and additionally of MODIS, MERIS and VIIRS data, are summarized in Table 3. Data related to cloud/ice flagging indicate that MSea followed by LSea and EIO, are the regions least affected by cloudiness. Specifically, MSea exhibits a rejection rate in the range of 53–63% across the different satellite data products. This significantly contrasts with the much higher values of 77–94% determined for ASea. It is also noted that NPO exhibits a rejection rate in the range of 72–84%.

In agreement with expectations, results from the analysis of high glint flagging vary from sensor to sensor and exhibit the lowest rejection rate in the range of 0–4% for SeaWiFS that benefits of tilt capability. The highest rejection rate that varies from 12 to 40% is observed for MERIS due to the small viewing swath in association with its early overpass time (however, when considering the generally lower rejection rate for clouds observed for MERIS, an interplay between glint and cloud flagging is likely to occur). Overall results indicate that, on average and regardless of the sensor, LSea appears to be the region least affected by glint while ASea located close to the equator is the most.

As expected, the high viewing angle flag largely affects SeaWiFS data with rejection rate of 30–44% due to the large viewing swath and its tilted view, while MERIS data are not at all affected due to the smaller swath.

When considering the combined effects of the previous three main flags, regardless of the sensor, MSea and LSea are the regions exhibiting the lowest rejection rates varying in the range of 65–74%. For comparison, NPO exhibits a rejection rate in the range of 85–89%.

As already anticipated the above results are definitely site and mission dependent. In fact, even assuming equivalent space sensors, the overpass time would have an impact on the daily percent observations due to glint and cloudiness affecting the same regions differently during the day (Feng and Hu, 2016).

### 5.2 Spatial homogeneity

Spatial homogeneity in the regions of interest is investigated through the coefficient of variation *C*_V_ (i.e., the ratio between standard deviation and the related mean) of the 5 × 5 Level-2 satellite derived *R*_rs_ values centered at the reference location. By restricting the analysis to cases in which all the 5 × 5 data elements are not affected by any of the SeaDAS Level-2 processing default flags, a region is heuristically considered spatially homogeneous when *C*_V_ is lower than 0.2 at the 443, 490 and 555 nm bands.

Results from the analysis of approximately 2000–3000 potential SeaWiFS observations per region over 5 years, are summarized in Table 4 (the number of observations varies from region to region and depends on latitude, onboard automatic recording over some sites and coverage provided by ground receiving stations). These results indicate that the Mediterranean regions (i.e., MSea and LSea) show the highest potential for matchups (i.e., observations not affected by default flags) with acceptance rates of 33 and 29%, respectively. In contrast ASea, NAO, CSea and NPO exhibit values ranging from 6 to 12%, while EIO reaches rates of 18%.

The test on homogeneity leads to an acceptance rate varying from 88% at NPO up to 97% at MSea with respect to cases not affected by the default flags. It is mentioned that the application of the more severe threshold *C*_V_ < 0.1 would decrease the previous acceptance rate to 79% at NPO and to 91% at MSea.

It is also recalled that the homogeneity test applied to *R*_rs_ data is mostly intended to ensure better comparability between in situ and satellite observations performed at very different spatial resolutions. Nevertheless, atmospheric optical properties around the SVC site may also exhibit spatial inhomogeneity. Its impact has been evaluated through the application of an additional homogeneity test to τ_a_(865) from the 5 × 5 data elements centered at the reference location. Results obtained from the application of the threshold *C*_V_ < 0.2 to *R*_rs_(443), *R*_rs_(490) and *R*_rs_(555), and of the additional threshold *C*_V_ < 0.3 to τ_a_(865), indicate a mean decrease of approximately 3% in the acceptance rate of matchups (the threshold of 0.3 applied to τ_a_(865) has been simply chosen to satisfy the much larger variation coefficients characterizing τ_a_(865) with respect to *R*_rs_(443), *R*_rs_(490) and *R*_rs_(555), as documented by the mean and standard deviation values later presented in Table 6). This result indicates a correlation between the spatial variability of *R*_rs_ and that of aerosol optical properties at the small scale considered, and suggests that the sole homogeneity test applied to *R*_rs_ may satisfy the need to flag cases affected by spatial inhomogeneity around SVC sites.

The analysis on SeaWiFS data is complemented by the determination of observations exhibiting a restricted range of sun zenith values (Table 4). Results obtained from the identification of those cases satisfying a threshold of θ_0_ ≤ 45° (versus a limit of 70° associated with the default processing flag) show that the sun zenith at NPO, CSea and ASea does not exceed the threshold applied, and also confirm an expected increase of threshold effects with latitude (with LSea being the most affected).

**Fig. 12:**
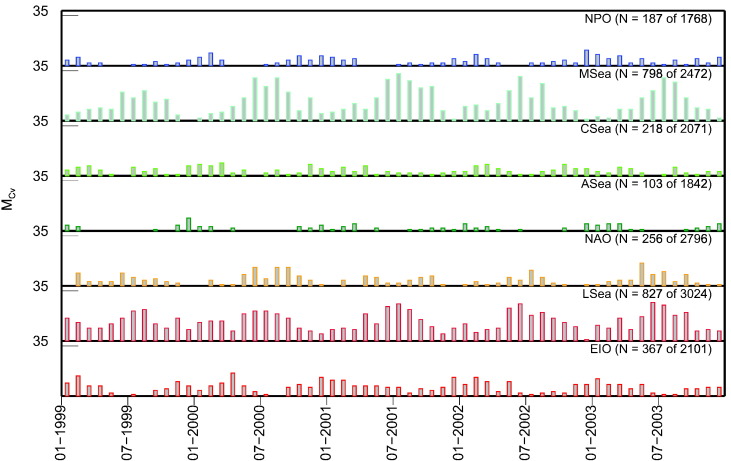
Number of SeaWiFS observations *M*_*CV*_ remaining after applying the default exclusion flags and passing the spatial homogeneity test (i.e., *C*_V_ < 0.2 for *R*_rs_ at the 443, 490 and 555 nm bands) for the 5-year period considered. Assuming no more than one daily observation, the maximum value of *M*_*CV*_ would not exceed 31 (for convenience the maximum value on the y-axes has been set to 35).

Fig. 12 illustrates the temporal distribution of SeaWiFS observations not affected by default flags and passing the spatial homogeneity test. Notable is the regular seasonal distribution of potential matchups at MSea, LSea, EIO and to a lesser extent at NAO, with peaks centered during local summer. Conversely, NPO, CSea and ASea exhibit a higher occurrence of data during winter likely explained by a more pronounced summer cloudiness (especially for CSea and ASea).

While analyzing Table 5 with the number of matchups for other missions, it should be noted the generally larger number of observations (i.e., *N*) of VIIRS available over 4 years when compared to those of MODIS over 5 years justified by the different viewing swaths (i.e., 3000 km versus 2300 km, respectively). Results in Table 5 show a lower number of non-flagged cases (*M*) with respect to those determined for SeaWiFS, a finding indicating a reduction in the capability of producing high quality matchups by MODIS, MERIS and VIIRS with respect to SeaWiFS. This can be explained by a lower number of available data (particularly for MERIS because of a smaller swath) and by a different performance of processing flags on data products from the various sensors.

Finally, the comparison of Table 4 with Table 5 indicates a generally higher number of cases removed by the homogeneity test when applied to SeaWiFS data as opposite to radiometric products from other ocean color sensors. This is very likely explained by a lower signal-to-noise ratio characterizing SeaWiFS radiometric data with respect to that of other sensors (Hu et al., 2012a).

## 6 Identification of prime SVC regions

The objective of this work is to identify the location of potential SVC sites suitable to support ocean color missions contributing to the creation of CDRs. While the analysis based on Level-3 data was expected to document the climatology of each marine region of interest, the actual identification of prime SVC sites is conditioned by the analysis of in situ and satellite matchups gathered from Level-2 products. Thus, in view of supporting the following investigation on relevance and also equivalence of SVC sites, results from the statistical analysis of marine/atmospheric 5-year SeaWiFS Level-2 daily full-resolution products are summarized in Table 6 for each region through mean *m* and standard deviation σ. These data provide an overview at the spatial scale of matchups on relevant bio-optical quantities and on their intra-annual variability (or conversely stability).

Values in Table 6 may show differences from those determined from the Level-3 monthly averaged data used to investigate the climatology of bio-optical atmospheric/marine quantities. These differences, in addition to a diverse spatial resolution and time binning, are also explained by the different quality control applied: more inclusive in the case of Level-3 products, driven by the objective to maximize the number of data products applicable for climatology analysis (i.e., retaining each valid Level-3 data element); and otherwise exclusive in the case of Level-2 products, finalized to the objective of preserving only those satellite observations applicable to the identification of high quality matchups (i.e., retaining only those cases for which all the 5 × 5 Level-2 data elements centered at the region of interest satisfy specific quality criteria).

The work of Zibordi et al. (2015) shows that the MOBY site in the NPO region, when compared to a number of alternative data sources, exhibits high capability to meet requirements for long-term stability essential for the creation of CDRs from multiple ocean color missions. This is explained by an outstanding effort to characterize, calibrate and maintain field radiometers in view of minimizing sources of uncertainties in derived in situ *L*_w_ data (Brown et al., 2007), together with a restricted range of illumination conditions, favourable marine/atmospheric optical properties and low intra-annual variability.

Results from 4, 5, definitely confirm the unique features of NPO with respect to any other region among those considered: maritime aerosols and oligotrophic waters exhibiting high intra-annual optical stability in addition to low sun zenith variations. Thus, the MOBY site remains a perfect candidate for SVC in support of climate applications, and can be considered a reference when looking for additional or alternative SVC sites relevant for the creation of CDRs.

### 6.1 Equivalence of measurement conditions across regions

It is recalled that equivalence of measurement conditions across marine regions is an element expected to minimize differences in *g*-factors regardless of the geographic location of the SVC site. However, despite the importance of this ideal requirement, the identification of multiple SVC sites may imply trading-off some of the criteria associated with the marine/atmospheric properties. For instance, with reference to results from the climatology analysis and also to data in Table 6, MSea followed by CSea and EIO are the regions that most compare with NPO in terms of intra-annual stability and mean values of the considered marine bio-optical quantities (i.e., *k*_d_(490) and *Chla*). When looking at *R*_rs_(555), CSea and EIO show variabilities (quantified by σ) lower than those observed at NPO, while ASea and MSea exhibit slightly higher values. At 412 nm, the lowest variability (better indicated by the coefficient of variation, σ/*m*, due to the wide range of *R*_*rs*_(412) values) shows the lowest values for NPO, followed by ASea, EIO, MSea and CSea.

When looking at the atmospheric optical quantities, the lowest intra-annual variability of both τ_a_(865) and α is observed at ASea and LSea. However, both regions exhibit values of α indicating contamination by continental aerosols more marked for LSea (and also seen for MSea). On the other hand, despite a lower intra-annual stability, CSea and EIO show mean values of α approaching those of NPO. It is however remarked that while CSea (see Fig. 9) is characterized by a relatively high seasonal variability of τ_a_(865), EIO (see Fig. 10) exhibits a more pronounced seasonal variability of α.

Finally, in addition to differences in sun zenith angles θ_0_ also given Table 6, elements worth mentioning are the ozone concentration *O*_3_ and the wind speed *W*_*S*_ across the considered marine regions. In fact, *O*_3_ strongly varies with latitude and may diversely affect the accuracy of the atmospheric correction process. Similarly, a different *W*_*S*_ may diversely perturb the reflectance of the sea surface and additionally the performance of measuring systems at sea (e.g., optical buoys).

Statistical analysis performed with ancillary data from SeaWiFS Level-2 products non flagged by the SeaDAS default exclusion flags, shows mean values of *O*_3_ varying from 253 and 259 DU at ASea and NPO, and up to 308 and 320 DU at NAO and LSea, respectively. Equivalent analysis performed on *W*_*S*_ ancillary data (originated by the US National Centers for Environmental Prediction (NCEP)) exhibits mean values in the range of 3–6 m s^− 1^ with σ generally within 1.5–2.5 m s^− 1^. More specifically, ASea, LSea and NPO show the lowest values (i.e., 3.2, 3.3, and 3.9 m s^− 1^, respectively), while CSea, MSea, NAO and EIO exhibit the highest (i.e., 4.6, 5.3, 5.5 and 6.0 m s^− 1^, still below the 12 m s^− 1^ threshold ensuring application of the SeaDAS whitecap correction).

Considering the previous differences among θ_0_, *O*_3_ and *W*_*S*_ from the various regions, a thorough evaluation of their impact on *g*-factors would require dedicated theoretical investigations, which are beyond the objective of this work. Because of this, the following investigation on best suitability of regions for SVC is restricted to *Chla*, τ_a_(865) and α.

### 6.2 Prime SVC regions

Assuming in situ *L*_w_ measurements are regularly available at each location considered, Table 7 presents the number of potential high quality matchups (i.e., applicable for SVC) between SeaWiFS and in situ data over a 5-year period, as identified through the application of very stringent criteria associated with oligotrophic conditions and clear marine atmosphere: *Chla* ≤ 0.1 μg l^− 1^ or τ_a_(865) ≤ 0.1 or α ≤ 1.0, or all of them. These thresholds have been chosen to tentatively reflect the statistical values determined for the NPO reference region and thus to implicitly identify cases characterized by oligotrophic conditions and maritime aerosols (Smirnov et al., 2003) as well as a small seasonal variability and a low marine bio-optical complexity. The use of the thresholds selected, implicitly favoring regions exhibiting high intra-annual stability, is thus expected to supersede any other index derived from the regional climatology of the relevant geophysical quantities.

Results show a dramatic decrease of the number of matchups when all quality criteria are applied. In particular, despite the low number of overall potential matchups (i.e., *M*_*CV*_ = 187) with respect to those available for other regions (e.g., *M*_*CV*_ = 798 for MSea or 828 for LSea), NPO exhibits the highest number of high quality matchups (i.e., *M*_Q1_ = 75). In addition to NPO, those regions showing an appreciable number of potential high quality matchups are MSea, CSea and EIO with *M*_Q1_ equal to 59, 48 and 42, respectively.

The number of potential high quality matchups obtained for NPO is fully supported by those determined from the application of MOBY data to SeaWiFS SVC. In fact, the number of 15 high quality matchups per year for NPO is comparable to the approximately 17 per year (i.e., 150 over a 9-year period) actually identified by Franz et al. (2007) for MOBY applying slightly different selection criteria. It is however recognized that the consistency of results across the various regions may be affected by geographical differences in the accuracy of data products. A specific case is that of *Chla* that is likely overestimated at MSea and LSea as a result of the application of global bio-optical algorithms (Morel and Gentili, 2009).

The numbers in Table 7, much smaller than the potential matchups determined solely applying the SeaDAS default exclusion flags in combination with the spatial homogeneity test, confirm the multi-annual effort generally required to produce mission specific *g*-factors qualified for the construction of CDRs.

Nevertheless, the need for a statistically significant number of matchups per mission (e.g., Franz et al., 2007), may suggest to increase their number by relaxing some of the thresholds applied to geophysical quantities. Results in Table 7 indicate that the potential for matchups at some regions can vastly increase through the application of less restrictive criteria. Examples are EIO and CSea, which exhibit typical *Chl*a values higher than those of regions like NPO or MSea (see Table 6). Thus, when relaxing the exclusion criteria and thus accepting mean values of *Chla* ≤ 0.2 μg l^− 1^ and also of τ_a_(865) ≤ 0.15, the number of potential matchups may massively increase for some regions (e.g., EIO). Nevertheless, the increase can be relatively moderate for others (e.g., NPO).

The choice of relaxing the selection criteria would, however, affect the desirable equivalence of multiple SVC sites. Besides, differences in the intra-annual variability of marine and atmospheric optical quantities at the diverse regions could unevenly impact the precision of *g*-factors across missions relying on different SVC sites.

Table 8 shows that the number of high quality potential matchups (i.e., *M*_Q1_) determined for MODIS, MERIS and VIIRS with the application of strict thresholds to geophysical quantities over the period considered, significantly varies from mission to mission, but in general exhibits regional values much lower than those determined for SeaWiFS. This result, fully supported by the number of matchups obtained through the sole application of default flags and homogeneity tests, may be additionally explained by the effects of thresholds in combination with systematic differences among data products. Still, regardless of the number of high quality matchups, notable is the consistency of M_Q1_ rates across the different regions. In fact, in all cases NPO (except for VIIRS) shows the best performance, followed by MSea, CSea and EIO in decreasing order. The very different number of potential matchups obtained for VIIRS at MSea when compared to those determined for MODIS is probably explained by the systematically lower mean regional values of α determined for the former (i.e., 1.04 ± 0.48) with respect to those computed for the latter (i.e., 1.34 ± 0.43).

In agreement with results from the analysis of SeaWiFS data, when relaxing the exclusion criteria for the selection of potential matchups for other sensor data by accepting mean values of *Chla* ≤ 0.2 μg l^− 1^ and of τ_a_(865) ≤ 0.15, their number (i.e., *M*_Q2_) largely increases for all sensors with respect to *M*_Q1_. Still these numbers are usually lower than those determined for SeaWiFS.

For completeness, the effects of applying the SeaDAS default limit value of 60° to the satellite zenith angle has also been investigated with the SeaWiFS dataset. The use of a 40° limit, that could further increase the quality of matchups and ideally make this quality more comparable across satellite sensors exhibiting different viewing swaths, was shown to lead to a reduction of *M*_*Q*1_ in the range of 45–75%. This large reduction appears to favor MSea with respect to NPO, but does not affect the overall relative ranking of the other marine regions.

## 7 Conclusions

Restating the fundamental importance of establishing SVC sites in regions that may benefit by logistical support from nearby islands or coastal locations, the study shows the difficulty in identifying regions located in different seas and characterized by ideal and likely equivalent measurement conditions: oligotrophic/mesotrophic waters and maritime aerosols in conjunction with low cloudiness, and high intra-annual stability and spatial homogeneity.

By relying on existing or potential fixed SVC sites (see Table 1), the study confirms the ideal location of the MOBY site associated with NPO. In fact, when looking at the marine optical properties (e.g., *K*_d_(490)) as derived from SeaWiFS Level-3 monthly average 24-th degrees products, NPO exhibits the least pronounced seasonal cycles. Additional regions characterized by high intra-annual stability are MSea and CSea, followed by EIO. It must be noted that the application of criteria favoring high intra-annual stability in the selection of SVC regions, may privilege low latitude with respect to higher latitudes regions, despite a lower number of overpasses and higher glint perturbations.

On the atmospheric side, Level-3 data exhibit the lowest and likely most stable values of τ_a_(865) at EIO, slightly increasing for LSea, NAO, MSea and NPO. Maritime aerosols empirically identified by mean values of α lower than 1, and also exhibiting high intra-annual stability, are determined at CSea and NPO. Still maritime aerosols, but affected by higher seasonal variability, are observed at EIO. MSea and LSea exhibit mean monthly values of α higher than 1 (i.e., 1.14 at MSea and 1.40 at LSea), but both characterized by high intra-annual stability.

From the analysis of Level-2 daily 1-km products, results indicate that MSea followed by LSea and EIO are the regions least affected by cloudiness with rejection rates due to cloud flagging in the range of 53–78% depending on region and satellite overpass. The spatial homogeneity, as determined from the analysis of the coefficient of variation *C*_V_ computed at the 443, 490 and 555 nm bands from the 5 × 5 values of *R*_rs_ centered at each location of interest, appears high for all the regions considered. Specifically, by solely considering observations passing tests from SeaDAS default exclusion flags, acceptance rates determined by *C*_V_ < 0.2 vary from 88% at NPO to 97% at MSea.

In view of drawing conclusions for the practical identification of SVC sites satisfying requirements for the creation of ocean color CDRs, the occurrence of ideal conditions equivalent to those observed at NPO has been investigated through the construction of potential in situ and satellite matchups. This has been performed using a number of strict quality tests applied to marine and atmospheric optical Level-2 data products passing the SeaDAS default flagging and spatial homogeneity checks, assuming that geographical differences in the accuracy of data products do not question basic conclusions. Summary results based on SeaWiFS Level-2 data and quality tests based on mean values of *Chla* ≤ 0.1 μg l^− 1^, τ_a_(865) ≤ 0.1 and α ≤ 1.0 from the 5 × 5 data elements centered at each region, indicate the possibility of obtaining 15 high quality matchups per year at NPO (consistent with the number of approximately 17 actually determined at the MOBY site), approximately 12 at MSea, 10 at CSea and 8 at EIO. The smaller number of potential high quality matchups at MSea, when compared to NPO, is explained by the rejection of cases affected by non-maritime aerosol. Conversely, in the case of CSea and EIO it is explained by rejections due to *Chla* > 0.1 μg l^− 1^. It must be emphasized that the previous number of matchups might however vary to some extent if a different atmospheric correction or regional bio-optical algorithms are adopted.

The study has also investigated the impact of lessening the criteria for the construction of matchups by using alternative thresholds such as *Chla* ≤ 0.2 μg l^− 1^ and τ_a_(865) ≤ 0.15. The increase in the number of potential matchups obtainable at regions like EIO or CSea is striking. However, in spite of the benefit of a larger number of matchups, the relaxation of selection criteria diminishes the equivalence of observation conditions among SVC sites. Yet, this reduced equivalence should not significantly impact the capability to satisfy the 5% requirement on *L*_w_ uncertainty. Contrarily, it could lessen the capability of SVC sites to meet the 0.5% per decade radiometric stability requirement due to a lower intra-annual environmental stability and likely a higher bio-optical complexity of the sites.

The same analysis performed with MODIS, MERIS and VIIRS Level-2 data provides a lower number of high quality matchups per year for all sensors, probably explained by systematic differences among products. Still the number of potential MODIS, MERIS and VIIRS matchups reflects the performance rate among marine regions determined with SeaWiFS data. It is also emphasized that the four ocean color missions considered offer a representative set of possible instrument and orbital characteristics. This suggests that conclusions from the study might be applicable to new or upcoming sensors such as the Ocean and Land Colour Instrument (OLCI) on-board the Sentinel-3 platforms.

In conclusion, the analysis on potential high quality matchups confirms the superior location of the MOBY site in the northern Pacific Ocean for SVC. While recognizing that no site is superior for all criteria reviewed in the analysis, it nonetheless suggests that the Eastern Mediterranean Sea near the Island of Crete exhibits best equivalence with NPO and could be considered as a further site for SVC complying with requirements for the creation of CDRs. Additional sites, even though exhibiting a lower capability of producing high quality matchups per year are the Caribbean Sea and the Indian Ocean near Rottnest Island.

It is finally restated that the previous findings are based on the analysis of optical properties of a limited number of SVC fixed sites already in place or under discussion, thus they do not exclude alternatives. In addition, it is also recognized that a theoretical investigation based on the application of actual atmospheric correction codes (e.g., SeaDAS) would likely help to better define thresholds for geophysical parameters satisfying SVC requirements.

## Figures and Tables

**Table 1:** Reference locations of the various marine regions considered in the following analysis.

Acronym	Region	Lon	Lat	Notes
SPG	South Pacific Gyre	− 125.0	− 25.0	Virtual site
NPO	North Pacific Ocean	− 157.8	19.4	Near the MOBY Site operated by NOAA
MSea	Mediterranean Sea	25.0	34.0	Potential site near Crete Island
CSea	Caribbean Sea	− 67.0	17.5	Potential site near Puerto Rico Islands
ASea	Arabian Sea	72.0	10.0	Near the Kavaratti Site operated by ISRO
NAO	North Atlantic Ocean	− 28.5	39.0	Potential site near Azores Islands
LSea	Ligurian Sea	8.0	43.5	Near the BOUSSOLE Site operated by LOV
EIO	Eastern Indian Ocean	114.5	− 32.0	Potential site near Rottnest Island

**Table 2:** Quantities investigated at each region of interest.

Acronym	Quantity	Relevance
*R*_rs_	Remote Sensing Reflectance	To address water type
*K*_d_(490) and *Chla*	Diffuse attenuation coefficient at 490 nm and Chlorophyll-a concentration^1^	To discuss climatology of marine bio-optical properties
τ_a_(865) and α	Aerosol optical thickness at 865 nm and Ångström exponent	To discuss climatology of atmospheric optical properties
*θ*_0_	Sun zenith	To address seasonal variability of illumination conditions

1 *Chla* determined with the Hu et al. (2012b) color index algorithm for values lower than approximately 0.25 μg l^− 1^ and the O'Reilly et al. (2000) band ratio for higher concentrations.

**Table 3:** Fraction of satellite observations (in %) affected by cloud/ice (*F*_*C*_), high glint (*F*_*G*_), and high viewing angle (*F*_*V*_) flags, or by all together (*Q*_*F*_), computed over the period considered for the various regions. The analysis relies on full resolution Level-2 data from the SeaWiFS, MODIS, MERIS and VIIRS ocean color sensors. Data were excluded when at least one of the 5 × 5 elements centered at the specific region was affected by the exclusion flag (s). The number of satellite observations per site and per sensor is given in Table 4, Table 5.

	SeaWiFS				MODIS				MERIS				VIIRS			
	*F*_*C*_	*F*_*G*_	*F*_*V*_	*Q*_*F*_	*F*_*C*_	*F*_*G*_	*F*_*V*_	*Q*_*F*_	*F*_*C*_	*F*_*G*_	*F*_*V*_	*Q*_*F*_	*F*_*C*_	*F*_*G*_	*F*_*V*_	*Q*_*F*_
NPO	77.4	1.8	35.5	84.8	83.5	14.9	15.3	86.4	71.6	33.9	0.0	82.2	80.9	11.7	31.6	88.6
MSea	53.5	0.2	31.6	65.4	63.3	12.3	17.0	72.9	53.5	25.6	0.0	68.1	56.1	10.0	28.5	73.4
CSea	74.7	4.4	37.7	84.2	78.2	18.5	14.3	82.4	68.9	37.0	0.0	84.9	74.0	13.8	31.8	85.3
ASea	88.6	3.3	42.0	92.6	91.1	21.2	14.3	92.5	76.8	40.2	0.0	87.8	93.6	14.9	27.9	96.4
NAO	84.5	0.0	29.8	87.9	83.6	11.8	16.0	87.5	72.5	25.2	0.0	80.4	83.4	8.4	26.7	89.1
LSea	61.9	0.0	30.3	69.1	67.4	3.7	21.0	72.6	61.6	12.0	0.0	66.4	66.2	3.2	25.0	73.5
EIO	65.1	0.8	44.0	79.2	65.5	13.6	12.4	74.3	67.6	19.7	0.0	76.3	62.1	11.1	28.9	78.4

**Table 4:** SeaWiFS Level-2 full-resolution data over a 5-year period (1999–2003) included in the statistical analysis and of those passing different quality tests for the various regions. *N* indicates the number of available observations, *M* is the number of cases remaining after applying the SeaDAS default exclusion flags, and *M*_*CV*_ indicates the number of cases that also passed the homogeneity test defined by a variation coefficient *C*_V_ < 0.2 determined from the 5 × 5 values of *R*_rs_ at the 443, 490 and 555 nm bands. Finally, *M*_*SZ*_ indicates the number of cases with respect to *M*, for which θ_0_ ≤ 45°.

	*N*	*M*	*M* vs *N* [%]	*M*_*CV*_	*M*_*CV*_ vs *M* [%]	*M*_*SZ*_	*M*_*SZ*_ vs *M* [%]
NPO	1768	212	12.0	187	88.2	211	99.5
MSea	2472	821	33.2	798	97.2	680	82.8
CSea	2071	242	11.7	218	90.1	242	100.0
ASea	1842	114	6.2	103	90.4	114	100.0
NAO	2796	274	9.8	256	93.4	211	77.0
LSea	3024	873	28.9	827	94.7	575	65.9
EIO	2101	382	18.2	367	96.1	309	80.9

**Table 5:** MODIS, MERIS and VIIRS full-resolution observations *N* for the various regions and various sensors over the period considered, together with those observations *M* not affected by the SeaDAS default exclusion flags, and those observations *M*_*CV*_ passing the homogeneity test.

	MODIS			MERIS			VIIRS		
	*N*	*M*	*M*_*CV*_	*N*	*M*	*M*_*CV*_	*N*	*M*	*M*_*CV*_
NPO	1708	132	132	760	118	117	1726	89	89
MSea	1922	447	441	888	223	221	1899	325	324
CSea	1591	202	202	740	107	105	1722	116	114
ASea	1589	69	66	737	73	71	1622	28	28
NAO	1977	158	158	991	110	105	2093	108	106
LSea	2165	482	477	1045	288	276	2272	397	394
EIO	1819	397	395	861	199	196	1910	266	266

**Table 6:** Mean *m* and standard deviation σ of SeaWiFS Level-2 non-flagged data products (*M*) utilized to complement the climatology analysis of the marine/atmospheric properties at the regions considered: *R*_*rs*_(412) and *R*_*rs*_(555) are units of sr^− 1^ × 10^− 3^, *k*_d_(490) in units of m^− 1^, *Chla* in units of μg l^− 1^, τ_a_(865) and α both dimensionless, and θ_0_ in units of degrees.

		*R*_*rs*_(412)		*R*_*rs*_(555)		*k*_d_(490)		*Chla*		τ_a_(865)		α		θ_0_	
	*M*	*m*	σ	*m*	σ	*m*	σ	*m*	σ	*m*	σ	*m*	σ	*m*	σ
NPO	212	12.9	1.45	1.54	0.29	0.027	0.004	0.07	0.01	0.07	0.04	0.88	0.40	29.5	12.3
MSea	821	9.48	2.38	1.51	0.33	0.029	0.006	0.09	0.03	0.08	0.05	1.22	0.41	26.8	14.2
CSea	242	8.64	2.31	1.54	0.23	0.033	0.009	0.13	0.07	0.08	0.05	0.69	0.42	23.8	12.7
ASea	114	6.57	1.19	1.62	0.30	0.043	0.011	0.19	0.11	0.11	0.05	1.14	0.29	24.2	9.3
NAO	274	6.46	2.11	1.68	0.41	0.047	0.020	0.25	0.22	0.06	0.04	1.09	0.45	31.7	14.2
LSea	873	5.09	2.03	1.65	0.41	0.051	0.020	0.28	0.23	0.07	0.04	1.45	0.37	38.7	15.9
EIO	382	7.51	1.81	1.53	0.25	0.036	0.008	0.15	0.05	0.05	0.03	0.76	0.55	28.3	14.7

**Table 7:** SeaWiFS Level-2 observations *M*_*CV*_ over the 5-year period considered, not affected by SeaDAS Level-2 default exclusion flags and passing the spatial homogeneity test, applied to investigate cases for which the 5 × 5 elements representing each region exhibit mean: *Chla* ≤ 0.1 μg l^− 1^, *Chla* ≤ 0.2 μg l^− 1^, τ_a_(865) ≤ 0.10, τ_a_(865) ≤ 0.15 and α ≤ 1.0. *M*_Q1_ indicates the number of potential high quality matchups identified through the application of combined tests on mean *Chla* ≤ 0.1 μg l^− 1^, mean τ_a_(865) ≤ 0.10 and mean α ≤ 1.0 (*M*_Q1_/year is the related number of potential high quality matchups per year). Differently, M_Q2_ indicates results from the application of combined tests on mean *Chla* ≤ 0.2 μg l^− 1^, mean τ_a_(865) ≤ 0.15 and mean α ≤ 1.0 (M_Q2_/year, indicates the related potential number of matchups per year).

	*M*_*CV*_	*Chla* ≤ 0.1	*Chla* ≤ 0.2	τ_a_(865) ≤ 0.10	τ_a_(865) ≤ 0.15	α ≤ 1.0	*M*_Q1_ (*M*_Q1_/year)	*M*_Q2_ (*M*_Q2_/year)
NPO	187	182	187	153	177	107	75 (15.0)	98 (19.6)
MSea	798	572	794	570	714	212	59 (11.8)	147 (29.4)
CSea	218	79	197	164	195	172	48 (9.6)	141 (28.2)
ASea	103	0	80	37	83	21	0 (0.0)	13 (2.6)
NAO	256	3	156	219	246	102	1 (0.2)	56 (11.2)
LSea	827	0	400	668	790	87	0 (0.0)	36 (7.2)
EIO	367	53	328	337	363	235	42 (8.4)	220 (44.0)

**Table 8:** MODIS, MERIS and VIIRS Level-2 observations, *M*_*CV*_, not affected by exclusion flags and passing the spatial homogeneity test for the various regions over the number of years considered. *M*_Q1_ indicates potential high quality matchups obtained by applying combined tests on mean *Chla* ≤ 0.1 μg l^− 1^, mean τ_a_(865) ≤ 0.1 and mean α ≤ 1.0 (*M*_Q1_/year, indicates the related potential number of high quality matchups per year). *M*_Q2_ refers to cases determined through combined tests on mean *Chla* ≤ 0.2 μg l^− 1^, mean τ_a_(865) ≤ 0.15 and mean α ≤ 1.0 (*M*_Q2_/year, indicates the related potential number of matchups per year).

	MODIS			MERIS			VIIRS		
	*M*_*CV*_	*M*_Q1_ (*M*_Q1_/year)	*M*_Q2_ (*M*_Q1_/year)	*M*_*CV*_	*M*_Q1_ (*M*_Q1_/year)	*M*_Q2_ (*M*_Q2_/year)	*M*_*CV*_	*M*_Q1_ (*M*_Q1_/year)	*M*_Q2_ (*M*_Q2_/year)
NPO	132	31 (6.2)	54 (10.8)	117	27 (5.4)	45 (9.0)	88	21 (5.3)	33 (8.3)
MSea	441	14 (2.8)	61 (12.2)	221	17 (3.4)	65 (13.0)	324	44 (11.0)	106 (26.5)
CSea	202	6 (1.2)	95 (19.0)	105	14 (2.8)	54 (10.8)	113	13 (3.3)	58 (14.5)
ASea	66	0 (0.0)	4 (0.8)	71	0 (0.0)	3 (0.6)	28	0 (0.0)	2 (0.5)
NAO	158	1 (0.2)	31 (6.2)	105	7 (1.4)	22 (4.4)	105	5 (1.3)	38 (9.5)
LSea	475	0 (0.0)	25 (5.0)	276	1 (0.2)	26 (5.2)	394	0 (0.0)	57 (14.3)
EIO	393	5 (1.0)	129 (25.8)	195	16 (3.2)	73 (14.6)	266	7 (1.8)	128 (32.0)
